# Elimination of Extracellular Adenosine Triphosphate for the Rapid Prediction of Quantitative Plate Counts in 24 h Time-Kill Studies against Carbapenem-Resistant Gram-Negative Bacteria

**DOI:** 10.3390/microorganisms8101489

**Published:** 2020-09-28

**Authors:** Yiying Cai, Jonathan J. Ng, Hui Leck, Jocelyn Q. Teo, Jia-Xuan Goh, Winnie Lee, Tse-Hsien Koh, Thuan-Tong Tan, Tze-Peng Lim, Andrea L. Kwa

**Affiliations:** 1 Department of Pharmacy, Singapore General Hospital, Singapore 169608, Singapore; cai.yiying@sgh.com.sg (Y.C.); LECK_Hui@moh.gov.sg (H.L.); jocelyn.teo.q.m@sgh.com.sg (J.Q.T.); goh.jia.xuan@sgh.com.sg (J.-X.G.); winnie.lee.h.l@sgh.com.sg (W.L.); 2 Department of Pharmacy, National University of Singapore, Singapore 117559, Singapore; Jonathan_Ng@cgh.com.sg; 3 Saw Swee Hock School of Public Health, National University of Singapore and National University of Health System, Singapore 117549, Singapore; 4 Department of Microbiology, Singapore General Hospital, Singapore 169856, Singapore; koh.tse.hsien@singhealth.com.sg; 5 SingHealth Duke-NUS Medicine Pathology Clinical Programme, Singapore 169608, Singapore; 6 Department of Infectious Diseases, Singapore General Hospital, Singapore 169856, Singapore; tan.thuan.tong@singhealth.com.sg; 7 SingHealth Duke-NUS Medicine Academic Clinical Programme, Singapore 169856, Singapore; 8 Emerging Infectious Diseases, Duke-NUS Medical School, Singapore 169857, Singapore

**Keywords:** time-kill studies, ATP bioluminescence, apyrase

## Abstract

Traditional in vitro time-kill studies (TKSs) require viable plating, which is tedious and time-consuming. We used ATP bioluminescence, with the removal of extracellular ATP (EC-ATP), as a surrogate for viable plating in TKSs against carbapenem-resistant Gram-negative bacteria (CR-GNB). Twenty-four-hour TKSs were conducted using eight clinical CR-GNB (two *Escherichia coli*, two *Klebsiella* spp., two *Acinetobacter baumannii*, two *Pseudomonas aeruginosa*) with multiple single and two-antibiotic combinations. ATP bioluminescence and viable counts were determined at each timepoint (0, 2, 4, 8, 24 h), with and without apyrase treatment. Correlation between ATP bioluminescence and viable counts was determined for apyrase-treated and non-apyrase-treated samples. Receiver operator characteristic curves were plotted to determine the optimal luminescence threshold to discriminate between inhibitory/non-inhibitory and bactericidal/non-bactericidal combinations, compared to viable counts. After treatment of bacteria with 2 U/mL apyrase for 15 min at 37 °C, correlation to viable counts was significantly higher compared to untreated samples (*p* < 0.01). Predictive accuracies of ATP bioluminescence were also significantly higher for apyrase-treated samples in distinguishing inhibitory (*p* < 0.01) and bactericidal (*p* = 0.03) combinations against CR-GNB compared to untreated samples, when all species were collectively analyzed. We found that ATP bioluminescence can potentially replace viable plating in TKS. Our assay also has applications in in vitro and in vivo infection models.

## 1. Introduction

Infections caused by carbapenem-resistant Gram-negative bacteria (CR-GNB) is a major public health problem worldwide [1]. Amidst the dearth of effective treatment options against CR-GNB, antibiotic combination therapy has emerged as one of the mainstay treatment options in Singapore against CR-GNB [2,3]. Time-kill studies (TKSs) provide information about the rate and extent of bacterial killing over time and are typically employed to guide antibiotic combination selection against CR-GNB [4]. One of the main drawbacks of TKSs is that they are laborious to complete, due to the need for enumeration of colony-forming units (CFUs) using viable plate counting [4]. Viable plate counting (also known as visual plate counting) is not only time-consuming due to the length of incubation time required for the bacteria to give rise to visible colonies but is also cumbersome to perform, especially when a large number of antibiotic combinations are tested.

Adenosine triphosphate (ATP) bioluminescence is a rapid bacterial enumeration method which uses firefly luciferase to convert chemical energy in the form of ATP into light energy that can be detected by a luminometer within a rapid turn-around time of 15–30 min [5]. The light intensity is directly proportional to the amount of ATP in the sample, which in turn is a surrogate of the measure of the total viable count [5]. To date, ATP bioluminescence has been used to quantify viable bacteria counts across multiple settings, including food production, water purification, and environmental testing [6,7,8]. Notably, in the field of rapid antimicrobial susceptibility testing, the use of ATP bioluminescence has been limited to semi-quantitative prediction of bacterial counts (i.e., inhibitory versus non-inhibitory) [9,10,11,12]. In previous susceptibility testing studies that attempted to use ATP bioluminescence to quantitatively predict bacterial counts upon exposure to antibiotics, correlation of ATP levels to bacterial counts was poor [13,14]. One postulation for the lack of correlation is that extracellular ATP (EC-ATP) is released during bacteria growth and upon bacterial cell lysis when exposed to antibiotics, leading to an overestimation of viable bacterial counts [15,16].

In light of the existing literature, we hypothesized that the determination of quantitative bacterial counts using ATP bioluminescence is limited by the presence of EC-ATP, and that eliminating EC-ATP will allow ATP bioluminescence to be accurately employed to quantify viable CR-GNB counts in combination TKSs. Hence, we developed a method to remove EC-ATP, and compared the ability of the ATP bioluminescence assay in quantifying CR-GNB in combination testing via TKSs before and after elimination of EC-ATP.

## 2. Materials and Methods

### 2.1. Bacterial Isolates

Eight clinical CR-GNB (two CR *Escherichia coli*, two CR *Klebsiella* spp., two CR *Acinetobacter baumannii*, and two CR *Pseudomonas aeruginosa*), collected as part of a national surveillance study in Singapore from 2009–2011, and four American Type Culture Collection (ATCC) strains (*E. coli* ATCC25922, *A. baumannii* ATCC19606, *P. aeruginosa* ATCC27853, and *Klebsiella pneumoniae* ATCC13883), were used. Genus identities of the clinical isolates were determined by the individual hospitals, using Vitek 2 ID-GN cards (bioMérieux Inc., Marcy-l’Étoile, France) and matrix-assisted laser desorption ionization time-of-flight (MALDI-TOF) mass spectrometry analysis. Susceptibility to carbapenems was determined by disk diffusion and interpreted using the Clinical and Laboratory Standards Institute (CLSI) guidelines [17]. The isolates were stored at −80 °C and fresh isolates were sub-cultured twice on 5% blood agar plates (Thermo Scientific, Waltham, MA, USA) for 24 h at 35 °C before each experiment.

### 2.2. Minimum Inhibitory Concentrations (MICs) and Mechanisms of Resistance

MICs of amikacin, polymyxin B, levofloxacin, meropenem, and tigecycline of the clinical isolates were determined using microbroth dilution panels (Trek Diagnostics, East Grinstead, UK) according to the manufacturer’s instructions. Susceptibility was defined using CLSI breakpoints, except for tigecycline, for which the breakpoints recommended by the US Food and Drug Administration (FDA) for Enterobacterales were used for *A. baumannii*, *E. coli*, and *Klebsiella* spp. [17].

Whole genome sequencing was employed to describe clonal typing and the presence of carbapenemases for clinical CR-GNB isolates. Briefly, genomic DNA was prepared from overnight bacterial cultures and extracted with the Qiagen Blood DNeasy kit (Qiagen Inc, Hilden, Germany). Genomic DNA was paired-end sequenced on a MiSeq system (Illumina Inc., San Diego, CA, USA), and reads had adaptors removed and were quality-trimmed using Trimmomatic software. The trimmed reads were used for de novo genome assembly using SPAdes (v.3.11.1). Sequence types (STs) were determined by performing a basic local alignment search tool (BLAST) search of the assembled contigs against a multilocus sequence typing (MLST) database (https://pubmlst.org/databases/). The SRST2 software tool was used to detect antimicrobial resistance genes using the ARGannot database [18].

### 2.3. Antimicrobial Agents and Reagents

A total of six antimicrobial agents, representing the major antibiotic classes with activity against Gram-negative bacteria, were employed. Amikacin was obtained from Discovery Fine Chemicals Ltd., Wimborne, UK. Aztreonam was obtained from Kemimac (s) Pte Ltd., Singapore. Levofloxacin was obtained from Daiichi Sankyo Co, Tokyo, Japan. Meropenem was obtained from Astra Zeneca Pharmaceuticals, Cambridge, UK. Polymyxin B was obtained from Sigma-Aldrich, St. Louis, MO, USA. Tigecycline was obtained from Wyeth Pharmaceuticals, Madison, NJ, USA. Stock solutions of all antibiotics except tigecycline were prepared in sterile water, aliquoted, and stored at −70 °C. Prior to each TKS, an aliquot of the drug was thawed and diluted to the desired concentration with freshly prepared cation-adjusted Mueller–Hinton broth (CA-MHB) (BD, Franklin Lakes, NJ, USA). Tigecycline in solution was freshly prepared and diluted to the desired concentration with freshly prepared CA-MHB before each TKS.

Apyrase from potatoes (A6535) was obtained from Sigma-Aldrich for removal of EC-ATP. Stock solutions of apyrase were prepared with sterile water (100 U/mL), aliquoted, and stored at −30 °C. Prior to each experiment, an aliquot of the apyrase was thawed and diluted to the desired concentration with normal saline. ATP was quantified using the BacTiter-Glo™ Microbial Viability Assay (Promega, Madison, WI, USA) prepared according to the manufacturer’s instructions. Briefly, 100 mL of the BacTiter-Glo™ buffer were transferred into an amber bottle containing the lyophilized substrate after equilibration to room temperature and employed within 24 h.

### 2.4. Experimental Conditions for Removal of EC-ATP

To determine the optimal concentration and treatment duration of apyrase to eliminate EC-ATP without affecting bacterial growth, we tested the ATCC reference strains (*E. coli* ATCC25922, *A. baumannii* ATCC19606, *P. aeruginosa* ATCC27853, and *K. pneumoniae* ATCC13883) against varying concentrations (0 (control), 0.25, 0.5, 1, 2 U/mL) and treatment durations (15, 30 min) of apyrase. Overnight cultures were diluted with pre-warmed CA-MHB and incubated at 35 °C until log-phase growth. The log-phase bacterial suspensions were diluted with CA-MHB to a final concentration of approximately 6log_10_ CFU/mL. One-milliliter bacterial suspensions were centrifuged (Eppendorf 5417R centrifuge, Hamburg, Germany) at 10,000× *g* for 5 min at 4 °C, reconstituted with sterile normal saline to their original volumes, and subjected to the different concentrations and durations of apyrase treatments at 35 °C. After apyrase treatment, each sample was washed twice with sterile normal saline by centrifugation at 10,000× *g* for 5 min at 4 °C, and samples were obtained in duplicate for (a) viable plating, (b) measurement of ATP (total), and (c) measurement of EC-ATP.

(a) *Viable plating.* Viable bacterial counts were quantified by depositing serial ten-fold dilutions of the broth sample onto Mueller–Hinton agar (MHA) plates (Thermo Scientific, Waltham, MA, USA), incubated at 35 °C for 18–24 h, and enumerated visually.
(b) *Measurement of ATP* (*total).* Bacterial ATP contents of the samples were quantified by the addition of 100 μL BacTiter-Glo™ assay reagent and bioluminescence intensities were recorded using the GloMax Integrated Luminescence System (Promega, Madison, WI, USA) with a 1 s integration time. Measurements were obtained in triplicate.
(c) *Measurement of EC-ATP.* To measure EC-ATP, the samples were filtered using an Acrodisc™ 25 mm syringe filter with a 0.2 μm Supor™ membrane (Pall Corporation, Port Washington, NY, USA) and the ATP content of the filtrate was measured using the GloMax Integrated Luminescence System. As intact bacterial cells were held within the filter membrane, the ATP bioluminescence measured in the filtrate reflected the EC-ATP content in each sample.

### 2.5. Relationship between ATP Bioluminescence and Viable Bacterial Counts

We described the relationship between bioluminescence (RLU) and colony-forming units (CFU) with and without apyrase treatment in the absence of antibiotics using the four ATCC strains. Overnight bacterial suspensions containing the ATCC strains were transferred to fresh CA-MHB and incubated until log-phase growth. The bacterial suspensions were then diluted with CA-MHB according to absorbance at 630 nm until reaching a final inoculum concentration of approximately 2–3log_10_ CFU/mL. The flasks were incubated in a shaking water bath at 35 °C and 0.5 mL samples were obtained serially at 0, 0.5, 1, 1.5, 2, 3, 4, 5, 6, 8, and 24 h. At each timepoint, duplicate samples of apyrase-treated samples (2 U/mL for 15 min at 35 °C) and apyrase-free controls were obtained for (a) viable plating and (b) measurement of ATP (total), as described above.

### 2.6. Time–Kill Studies

Against CR *E. coli*, CR *Klebsiella* spp., and CR *A. baumannii*, TKSs were performed for amikacin, levofloxacin, meropenem, polymyxin B, and tigecycline individually, as well as for the single most promising combination regimen based on in vitro findings in previous studies on the isolates [19,20,21,22,23]. For CR *P. aeruginosa*, TKSs were performed for aztreonam instead of tigecycline, as *P. aeruginosa* is intrinsically resistant to tigecycline. The simulated steady-state free concentrations of the antibiotics and their corresponding representative doses are shown in Supplementary Table S1 [24,25,26,27,28,29]. To perform the TKSs, overnight bacterial cultures were prepared using CA-MHB and incubated at 35 °C until log-phase growth. The bacteria were further diluted and 23 mL transferred to sterile flasks each containing 1 mL of drug(s) at 23 times the target concentration, to a final inoculum concentration of approximately 5log_10_ CFU/mL (1 × 10^5^ CFU/mL–5 × 10^5^ CFU/mL). The flasks were incubated in a shaking water bath at 35 °C, and serial samples were obtained in duplicate at 0, 2, 4, 8, and 24 h. At each timepoint, duplicate samples of apyrase-treated samples (2 U/mL for 15 min at 35 °C) and apyrase-free controls were obtained for (a) viable plating and (b) measurement of ATP (total), as described above. The limit of detection for this viable colony count method, achieved by plating 50 μL of each dilution onto MHA plates, was 1.30log_10_ CFU/mL.

### 2.7. Data Analysis

All data were analyzed using R version 3.6.1 software (R Foundation for Statistical Computing, Vienna, Austria). For each specimen and antibiotic combination, background RLU values (obtained from blank CA-MHB) were first subtracted to obtain log_10_-corrected RLU values at each timepoint. Receiver operating characteristic (ROC) curve analyses were carried out using the log_10_-corrected RLU values to determine the sensitivity and specificity of ATP bioluminescence in distinguishing between (1) inhibitory and non-inhibitory and (2) bactericidal and non-bactericidal antibiotic treatment regimens, as determined by viable plating in apyrase-treated and non-apyrase-treated samples, for all CR-GNB and individual species. Comparisons of the area under the ROC curve between apyrase-treated and non-apyrase-treated samples were conducted using “pROC” in R [30]. Pearson’s correlation coefficients were tabulated for log_10_-corrected RLU values (log_10_ RLU/100 μL) versus viable counts (log_10_ CFU/mL) for all CR-GNB and for each species. Outliers were omitted by visually gauging the clustering of data points. Comparisons of correlation coefficients between apyrase-treated and non-apyrase-treated samples were conducted using “cocor” in R for dependent non-overlapping samples [31]. A final two-tailed *p*-value of <0.05 was considered to be significant.

## 3. Results

### 3.1. Characteristics of the CR-GNB Isolates

The MICs and genotypic characteristics of the eight CR-GNB isolates are shown in Table 1. All isolates were resistant to amikacin, aztreonam, levofloxacin, cefepime, meropenem, and imipenem. Polymyxin B MICs ranged from 1–4 mg/L. No interpretative standards are provided by the CLSI for Enterobacterales or *P. aeruginosa* against tigecycline; tigecycline MICs for the clinical isolates ranged from 0.25–≥ 32 mg/L [17]. Out of the eight clinical isolates, seven harbored genes encoding carbapenemases (Table 1). Both *A. baumannii* isolates harbored *bla*_OXA-23_ and *bla*_OXA-51_, with insertion sequence IS*Aba*1 upstream of these genes. All four CR Enterobacterales isolates harbored carbapenemases—two isolates (EC195 and KP44) harbored *bla*_NDM_, one isolate (EC196) harbored *bla*_OXA-48_ and one isolate (KP215) harbored *bla*_KPC-2_. One CR *P. aeruginosa* isolate (PA4) harbored *bla*_IMP-1_.

### 3.2. Experimental Conditions for Removal of EC-ATP

**Figure 1:**
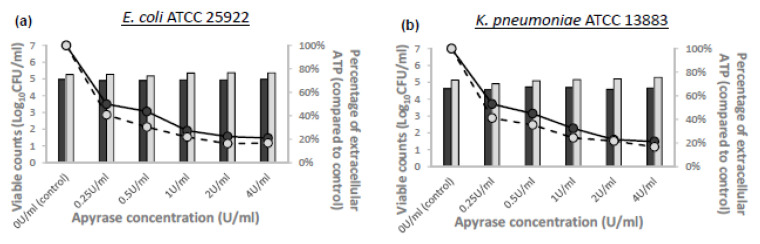

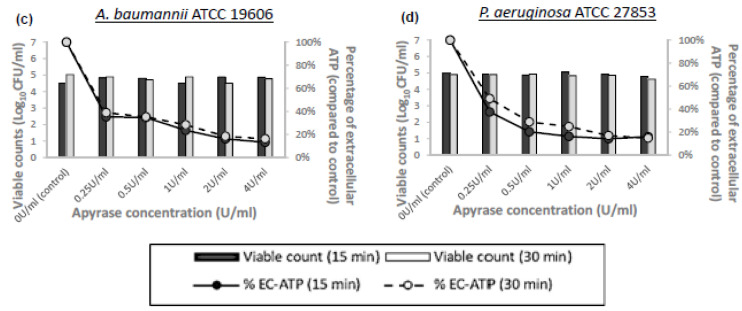
Effects of varying apyrase concentrations (U/mL) and incubation times on average viable count (log_10_CFU/mL) and total bacterial ATP in (**a**) *E. coli* ATCC25922, (**b**) *K. pneumoniae* ATCC13883, (**c**) *A. baumannii* ATCC19606, and (**d**) *P. aeruginosa* ATCC27853.

The viable bacterial counts and percentages of EC-ATP (compared to untreated bacteria) of the apyrase-treated samples at different apyrase concentrations and incubation durations for the four ATCC reference strains are shown in Figure 1. As shown, increasing apyrase concentrations up to 4 U/mL at 15 min or 30 min did not have an adverse effect on viable bacterial counts compared to non-apyrase-treated samples for all ATCC reference strains. An appreciable reduction in bacterial EC-ATP was observed when the apyrase concentration was increased from 0.25 U/mL to 2 U/mL at an incubation period of 15 min; increasing apyrase concentrations beyond 2 U/mL did not result in further reduction of EC-ATP. Increasing the incubation period with apyrase from 15 min to 30 min did not result in an appreciable reduction in bacterial EC-ATP for all apyrase concentrations. Hence, we selected the final experimental conditions of 2 U/mL apyrase for 15 min at 37 °C for the TKSs.

### 3.3. Relationship between ATP Bioluminescence and Viable Bacterial Counts

In the absence of antibiotic treatment, the ATP bioluminescence assay (log_10_ RLU/100 μL) displayed a linear relationship with viable counts (log_10_ CFU/mL) in the operating range of approximately 2–7log_10_ CFU/mL, with apyrase (*r*^2^ range: 0.90–0.98) and without apyrase treatment (*r*^2^ range: 0.95–0.99) (Supplementary Figure S1a,b). The lower limit of detection was approximately 2log_10_ RLU/100 μL, which corresponded to approximately 2–3log_10_ CFU/mL for all strains; below this, bacterial RLU readings were confounded by the background RLU readings in sterile MHB. The maximum limit of detection approximately 8log_10_ RLU/100 μL, which corresponded to approximately 8–9log_10_ CFU/mL for all strains.

### 3.4. Time-Kill Studies (TKSs)

#### 3.4.1. Viable Counts

The viable counts obtained in the TKSs are described in Supplementary Figure S2. Levofloxacin alone and tigecycline alone were not bactericidal or at least inhibitory against any of the CR-GNB strains at 24 h. Similarly, aztreonam alone was neither bactericidal nor at least inhibitory against either of the CR *P. aeruginosa* strains at 24 h. Meropenem alone exhibited inhibitory activity against 1/8 CR-GNB (PA23 against meropenem at 24 h = 3.26log_10_ CFU/mL) at 24 h. Amikacin alone was at least inhibitory against 3/8 CR-GNB (AB10, PA4, PA23) at 24 h; of these, amikacin was bactericidal against one out of the three CR-GNB strains (AB10 against amikacin at 24 h = 1.60log_10_ CFU/mL). Polymyxin B alone exhibited inhibitory and bactericidal activity against 5/8 and 4/8 CR-GNB strains, respectively; in the remaining three CR-GNB strains that were not at least inhibited (KP44, AB17, PA23), polymyxin B was bactericidal at 2–4 h, but regrowth occurred and reached more than 5log_10_ CFU/mL at 24 h. Six out of the eight two-antibiotic combinations were at least inhibitory against the CR-GNB strains at 24 h—polymyxin B + tigecycline was bactericidal against EC195, EC196, and AB17; polymyxin B + levofloxacin was bactericidal against KP215; and polymyxin B + amikacin was bactericidal against PA4 and PA23.

#### 3.4.2. Prediction of Inhibitory/Non-Inhibitory and Bactericidal/Non-Bactericidal Activity Using ATP Bioluminescence

**Figure 2:**
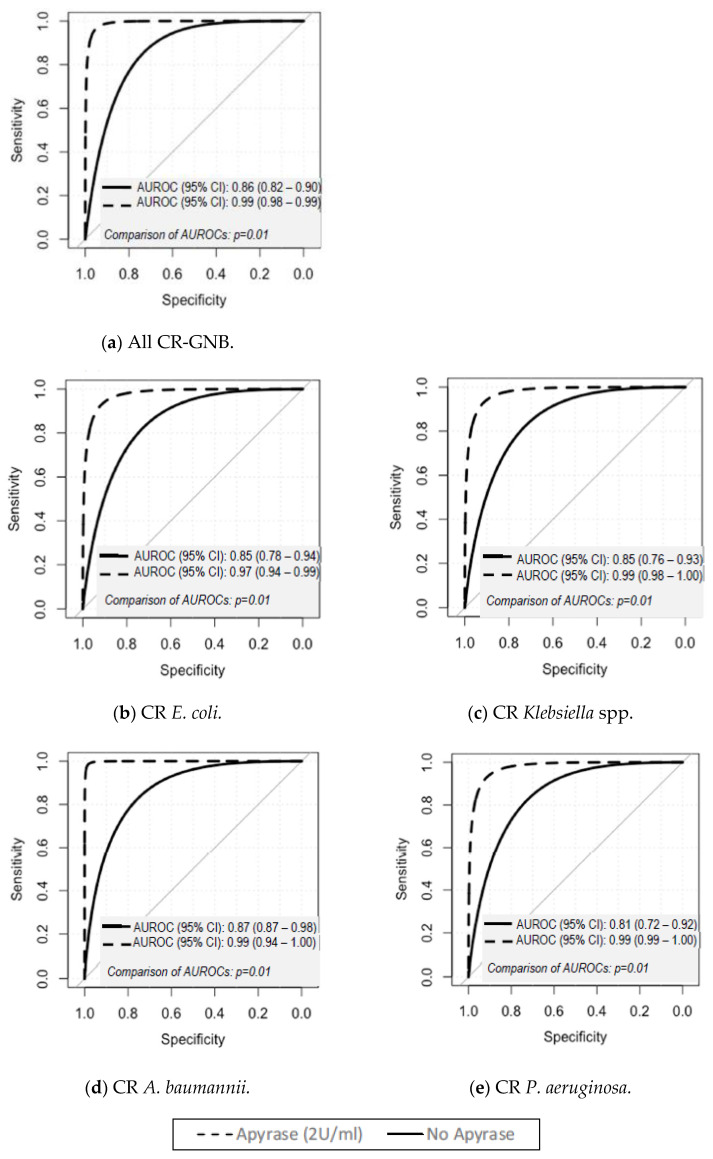
Receiver operating curves (ROC) plots for distinguishing between inhibitory and non-inhibitory activity at different timepoints for (**a**) All CR-GNB, (**b**) CR *E. coli*, (**c**) CR *Klebsiella* spp., (**d**) CR *A. baumannii*, and (**e**) CR *P. aeruginosa* with and without apyrase (2 U/mL) in 24 h time–kill studies (TKSs).

The ROC curves for the prediction of inhibitory and non-inhibitory activities using ATP bioluminescence assays with and without removal of EC-ATP for all CR-GNB and for each species are shown in Figure 2. When all CR-GNB organisms were collectively analyzed, the unweighted accuracies for the ATP bioluminescence assays with and without removal of EC-ATP were 95.8% and 79.4%, respectively (comparison of ROC curves: *p* < 0.01). The unweighted accuracies of the bioluminescence assay were also higher in apyrase-treated samples (range of unweighted accuracies: 92.2–98.4%) than in non-apyrase-treated samples (range of unweighted accuracies: 76.9–81.5%) for each species.

**Figure 3:**
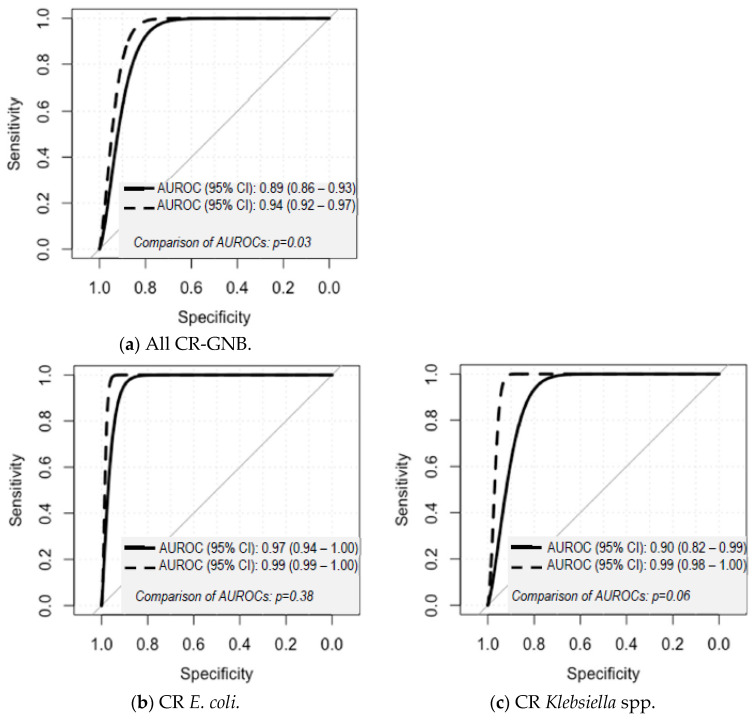

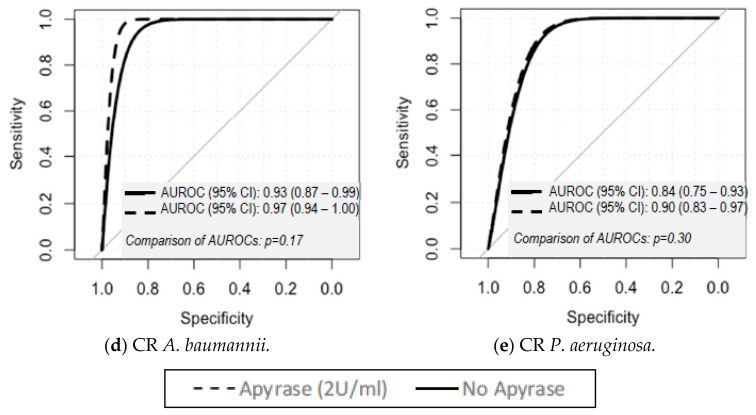
ROC plots for distinguishing between bactericidal and non-bactericidal activity at different timepoints for (**a**) All CR-GNB, (**b**) CR *E. coli*, (**c**) CR *Klebsiella* spp., (**d**) CR *A. baumannii*, and (**e**) CR *P. aeruginosa* with and without apyrase (2 U/mL) in 24 h TKSs.

The ROC curves for the prediction of bactericidal and non-bactericidal activities using ATP bioluminescence assays with and without removal of EC-ATP for all CR-GNB and for each species are shown in Figure 3. When all GNB organisms were collectively analyzed, the unweighted accuracy for the ATP bioluminescence assays with EC-ATP was significantly higher in apyrase-treated samples (90.2%) compared to non-apyrase-treated samples (80.6%) (comparison of ROC curves: *p* = 0.03). However, we did not observe significant improvement in the apyrase-treated samples (range of unweighted accuracies: 84.7–97.2%) compared to non-apyrase-treated samples (range of unweighted accuracies: 83.9–92.9%) when each species was individually analyzed.

#### 3.4.3. Correlation between ATP Bioluminescence and Viable Counts

**Figure 4:**
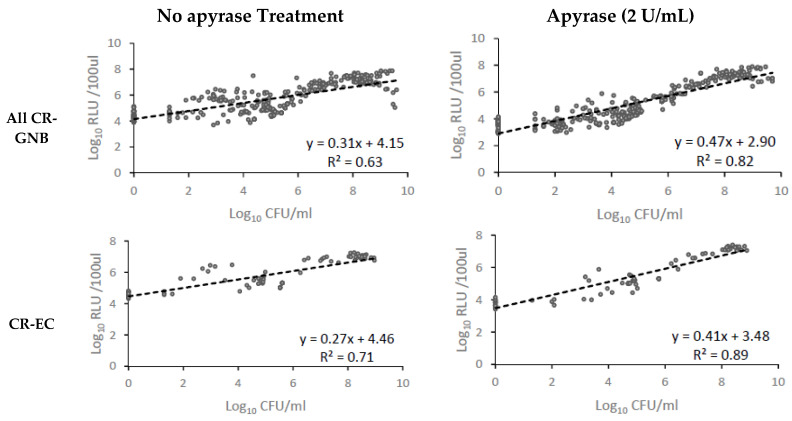

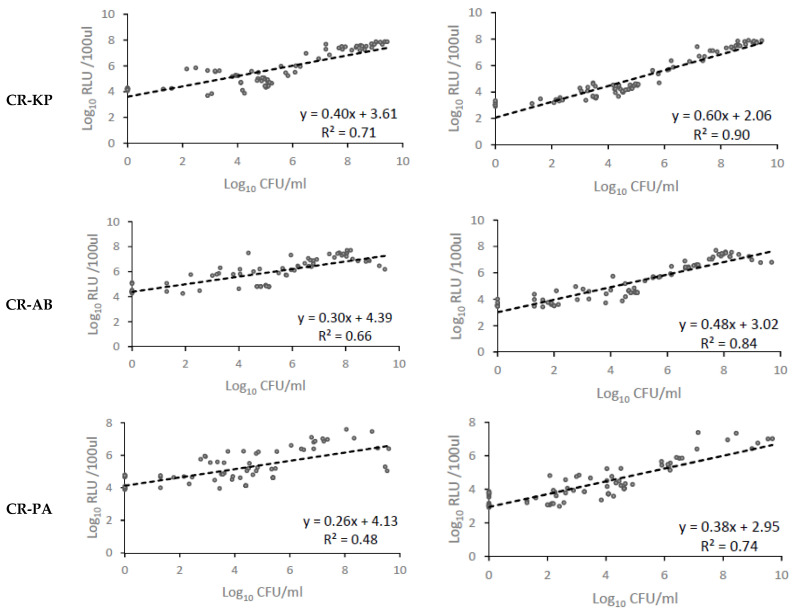
Correlation between viable counts (log_10_CFU/mL) and ATP bioluminescence (log_10_RLU/100 μL) in samples with and without apyrase (2 U/mL) in 24 h time–kill studies.

Table 2 summarized the coefficient of determination (*r*^2^) between ATP bioluminescence (log_10_ RLU/100 μL) and viable plate counts (log_10_ CFU/mL) in the TKS, overall and by GNB species, with and without removal of EC-ATP with apyrase, while the individual scatter plots are shown in Figure 4. Significant improvement in the correlation to viable counts was observed for all organisms in apyrase-treated samples compared to untreated samples (*r* = 0.79 versus *r* = 0.90; *p* < 0.01). The correlation was also significantly better when each CR-GNB species was separately analyzed, with the coefficient of determination ranging from 0.74–0.90 in apyrase-treated samples, compared to 0.48–0.71 in non-apyrase-treated samples. The actual viable counts and viable counts predicted using ATP bioluminescence with and without removal of EC-ATP across a 24 h TKS after exposure to various antibiotics are shown in Supplementary Figure S3. As shown, after removal of EC-ATP, ATP bioluminescence predicted antibiotic regimens that are at least inhibitory at 24 h, and provided information on the dynamics of bacterial killing over time with a reasonably high degree of accuracy.

## 4. Discussion

In this study, we applied ATP bioluminescence to single-drug and two-drug antibiotic combination TKSs against CR-GNB, before and after removal of EC-ATP. We found that removal of EC-ATP using apyrase significantly improved the accuracy of ATP bioluminescence in predicting both inhibitory and bactericidal antibiotic regimens against CR-GNB. In addition, ATP bioluminescence corelated well to quantitative bacteria counts after removal of EC-ATP and can be used to provide information on the bacterial killing dynamics over time in TKSs. Potentially, our ATP bioluminescence method eliminates the need for conventional viable plating methods in TKSs. This will reduce the manpower needed and improve turn-around time and allow for a larger number of antibiotic combinations to be tested.

Our study contributes to the body of research on the development of ATP bioluminescence assays for assessing antimicrobial susceptibility and antibiotic combination testing in bacteria [9,11,12,13,32,33]. In a recent study, Matsui et al. developed a rapid ATP bioluminescence-based test for detecting levofloxacin resistance in positive blood culture bottles, thereby reducing the turn-around time for antibiotic susceptibility testing [32]. In another recent publication, our study team developed an ATP-bioluminescence assay to identify antibiotic combinations that are at least inhibitory against CR-GNB across a wide array of antibiotic combinations, within a rapid turn-around time of 6 h [11]. While the findings of these studies have reinforced the potential of ATP bioluminescence to replace traditional susceptibility testing methods, studies to date have only been able to employ ATP bioluminescence to predict viable bacteria counts in a semi-quantitative manner. In a study in 2008, Ivancic et al. described a bioluminescent approach for the rapid determination of the antimicrobial susceptibility profiles of uropathogens in clinical urine specimens [13]. While their assay demonstrated high unweighted accuracy when thresholds for individual species were employed, correlation between ATP bioluminescence and actual quantitative bacterial counts appeared to be poor. In another study by Boswell et al., the authors attempted to correlate ATP bioluminescence to viable plate counts; in the study, there was a poor correlation between the two methods after antibiotic exposure, even after treatment with 0.04% apyrase [14].

EC-ATP levels found in bacterial supernatant are contributed to by a two-fold mechanism: firstly, even in the absence of antibiotics, EC-ATP is produced by many bacterial species during growth and serves a role in bacterial physiology; secondly, in the presence of antibiotics, ATP is released from dead and lysed bacteria, which can result in high levels of EC-ATP in the supernatant [15,34]. Apyrase is an enzyme with adenosine triphosphatase and adenosine diphosphatase activities, and has been widely applied in previous studies for the removal of EC-ATP [16,32,35,36]. Similar to the findings of our study, Sakakibara et al. demonstrated that apyrase effectively removes EC-ATP with minimal effect on intracellular ATP or bacterial growth [16]. As a wide array of varying concentrations and incubation times with apyrase were employed across different studies, we subjected different species of reference ATCC strains to varying apyrase concentrations and incubation times to identify the ideal apyrase concentration and incubation time in our study. To determine the percentage of EC-ATP remaining in the samples after apyrase treatment, we measured the ATP bioluminescence of the bacterial supernatant after passing the samples through a 0.2 μm syringe filter as a surrogate for EC-ATP; as intact bacterial cells are retained with the filter membrane, any ATP remaining in the supernatant will represent EC-ATP within the samples. From our study, we observed that the effect of apyrase on the removal of EC-ATP plateaued at approximately 80%; we hypothesize that our observation may be due to EC-ATP being continually produced by bacteria during growth [15].

Prior to the removal of EC-ATP, the semi-quantitative predictions of inhibitory antibiotic regimens in TKSs using ATP were only fair [area under the ROC curves (AUROC) for each species: 0.81–0.87] but were significantly improved after the removal of EC-ATP (AUROC for each species: 0.97–0.99). In contrast, semi-quantitative predictions of bactericidal antibiotic regimens in TKSs using ATP without EC-ATP removal were already good for most CR-GNB species (AUROC for each species except *P. aeruginosa*: 0.90–0.93). This may explain why we did not observe significant improvement in semi-quantitative predictions of bactericidal activity for each species after treatment with apyrase (AUROC for each species except *P. aeruginosa*: 0.97–0.99). In the quantitative analysis, ATP bioluminescence after EC-ATP removal appeared to correlate well to viable plate counts within the range of 2–8log_10_ CFU/mL and can be used to elucidate the rate and extent of the bacterial killing dynamics over time. Higher coefficients of determination were obtained when the different CR-GNB species were separately analyzed; this suggested that there may be differences in basal ATP content between different bacterial species [5]. Hence, it is desirable to determine individual calibration curves at the species level. Moving forward, external validation of the calibration curves with a larger number of CR-GNB isolates and antibiotic combinations will be prudent.

The results of our study suggest that ATP bioluminescence can potentially replace the process of viable plating in the conduction of in vitro studies. In future studies, we believe that the accuracy of our assay can be further improved. It has been hypothesized that filaments or protoplasts, which are osmotically fragile cells with high ATP content induced by exposure to beta-lactam antibiotics, may contribute to the inconsistencies in the relationship between ATP bioluminescence and viable plate counts [37,38]. Wheat et al. proposed using a growth media with lower osmolality to remove filaments, while Hattori et al. developed a method for selectively removing filamentous ATP from the filaments before bioluminescence measurement [38,39]. We hope to explore both methods in future studies. In addition, viable but non-dividing persister cells, which produce basal ATP but do not form colony-forming units in viable plating, may have contributed to the inaccuracies of our ATP bioluminescence assay [40]. We aim to investigate persister development using flow cytometry in our future studies.

## 5. Conclusions

To date, in vitro TKSs remain the mainstay for determining effective antibiotic combinations against CR-GNB. Unfortunately, the elucidation of viable bacterial counts, which is a fundamental procedure in the conduction of TKSs, is a slow and labor-intensive process. Our ATP bioluminescence assay with EC-ATP removal can potentially replace the process of viable plating with high accuracy. Our assay has applications beyond in vitro TKSs, such as for in vitro hollow fiber infection models, as well as in vivo animal studies. Future studies will be conducted to further improve and expand the utility of our assay.

## Figures and Tables

**Table 1:** Minimum inhibitory concentrations (mg/L) and resistance mechanisms of the eight carbapenem resistant Gram-negative bacteria (CR-GNB) clinical isolates.

Species	*E. coli*		*Klebsiella* spp.		*A. baumannii*		*P. aeruginosa*	
Strain no.	EC195	EC196	KP44	KP215	AB10	AB17	PA4	PA23
Molecular mechanisms of resistance								
Carbapenemases	NDM-1	OXA-48	NDM-1	OXA-181	OXA-23 OXA-51	OXA-23 OXA-51	IMP-1	-
Minimum inhibitory concentrations (MICs)								
Amikacin	≥128	≥128	≥128	≥128	32	≥128	32	≥128
Aztreonam	≥64	≥64	≥64	≥64	≥64	≥64	16	≥64
Cefepime	≥64	≥64	≥64	≥64	≥64	≥64	≥64	≥64
Levofloxacin	≥64	≥64	≥64	≥64	≥64	16	32	≥64
Imipenem	≥64	≥64	≥64	≥64	32	≥64	16	32
Meropenem	≥64	≥64	≥64	≥64	32	≥64	16	16
Polymyxin B	1	1	4	1	1	1	2	1
Tigecycline	0.25	0.5	2	4	≥32	2	8	16

**Table 2:** Comparison of coefficient of determination between viable counts (log_10_CFU/mL) and ATP bioluminescence (log_10_RLU/100 μL) with and without apyrase (2 U/mL) in 24 h time-kill studies.

Organism	Coefficient of Determination (*r*^2^)		*p*-Value
	No Apyrase	Apyrase	
All CR-GNB	0.63	0.82	<0.01
CR *E. coli*	0.71	0.89	<0.01
CR *Klebsiella* spp.	0.71	0.90	<0.01
CR *A. baumannii*	0.66	0.84	<0.01
CR *P. aeruginosa*	0.48	0.74	<0.01

## Supplementary Materials

The following are available online at https://www.mdpi.com/2076-2607/8/10/1489/s1, Figure S1: Relationship between ATP bioluminescence and viable plate counts for ATCC reference strains in the absence of antibiotics, Figure S2: Summary of viable bacterial counts for (a) CR *E. coli*, (b) CR *Klebsiella* spp., (c) CR *A. baumannii*, and (d) CR *P. aeruginosa* in 24 h TKSs, Figure S3: 24 h TKS curves of actual viable counts (solid line) versus viable counts predicted using ATP bioluminescence (dotted line) for EC195 for (a) polymyxin B, (b) meropenem, (c)levofloxacin, (d) amikacin, (e) tigecycline, and (f) polymyxin + tigecycline, Table S1: Antibiotic concentrations used in TKSs and corresponding doses for simulated concentrations.
