# Supplementary material for: Elimination of Extracellular Adenosine Triphosphate for the Rapid Prediction of Quantitative Plate Counts in 24 h Time-Kill Studies against Carbapenem-Resistant Gram-Negative Bacteria
Source: Microorganisms. 2020 Sep 28;8(10):1489. doi: 10.3390/microorganisms8101489 (PMC7599598; doi:10.3390/microorganisms8101489)

Supplementary Appendix

Supplementary Table S1. Antibiotic concentrations used in TKS and corresponding doses for simulated concentrations

Antibiotic	Conc. (mg/l)	Dosing regimens	Ref.
Amikacin	65	20mg/kg IBW	[24]
Aztreonam	24	8g every 24h (infused over 24h)	[25]
Levofloxacin	8	750mg every 24 hours	[26]
Meropenem	20	2g every 8 hours (infused over 3 hour)	[27]
Polymyxin B	2	30,000IU/kg/day or at least 1MU every 12 hours	[28]
Tigecycline	2	100mg every 12 hour	[29]

Supplementary Figure S1: Relationship between ATP bioluminescence and viable plate counts for ATCC reference strains in absence of antibiotics

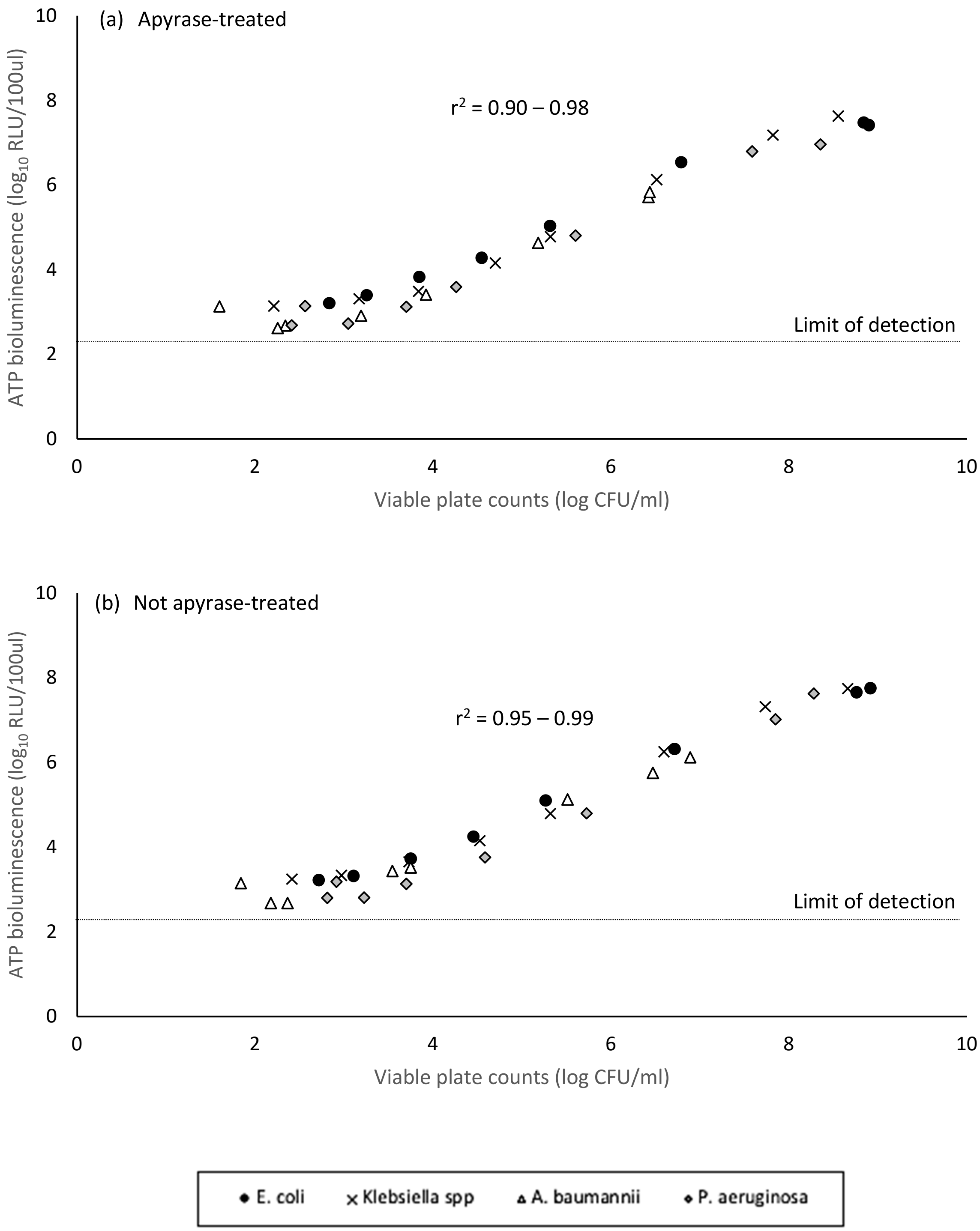

Supplementary Figure S2. Summary of viable bacterial counts for (a) CR *E. coli*, (b) CR *Klebsiella* spp, (c) CR *A. baumannii*, and (d) CR *P. aeruginosa* in 24h TKS

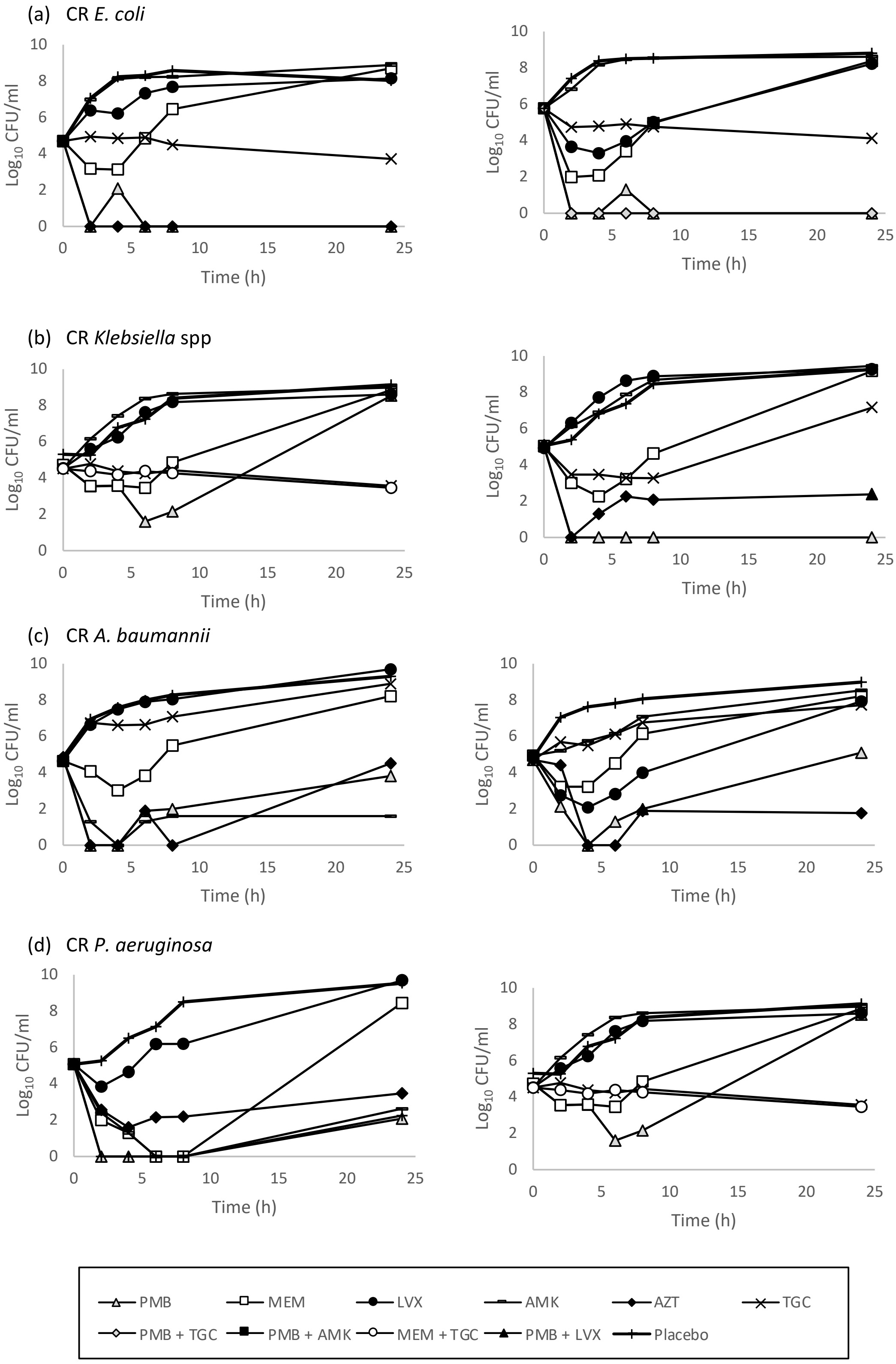

Supplementary Figure S3. 24h TKS curves of actual viable counts versus viable counts predicted using ATP bioluminescence with and without apyrase treatment for EC195 for (a) polymyxin B, (b) meropenem, (c)levofloxacin, (d) amikacin, (e) tigecycline, and (f) polymyxin + tigecycline

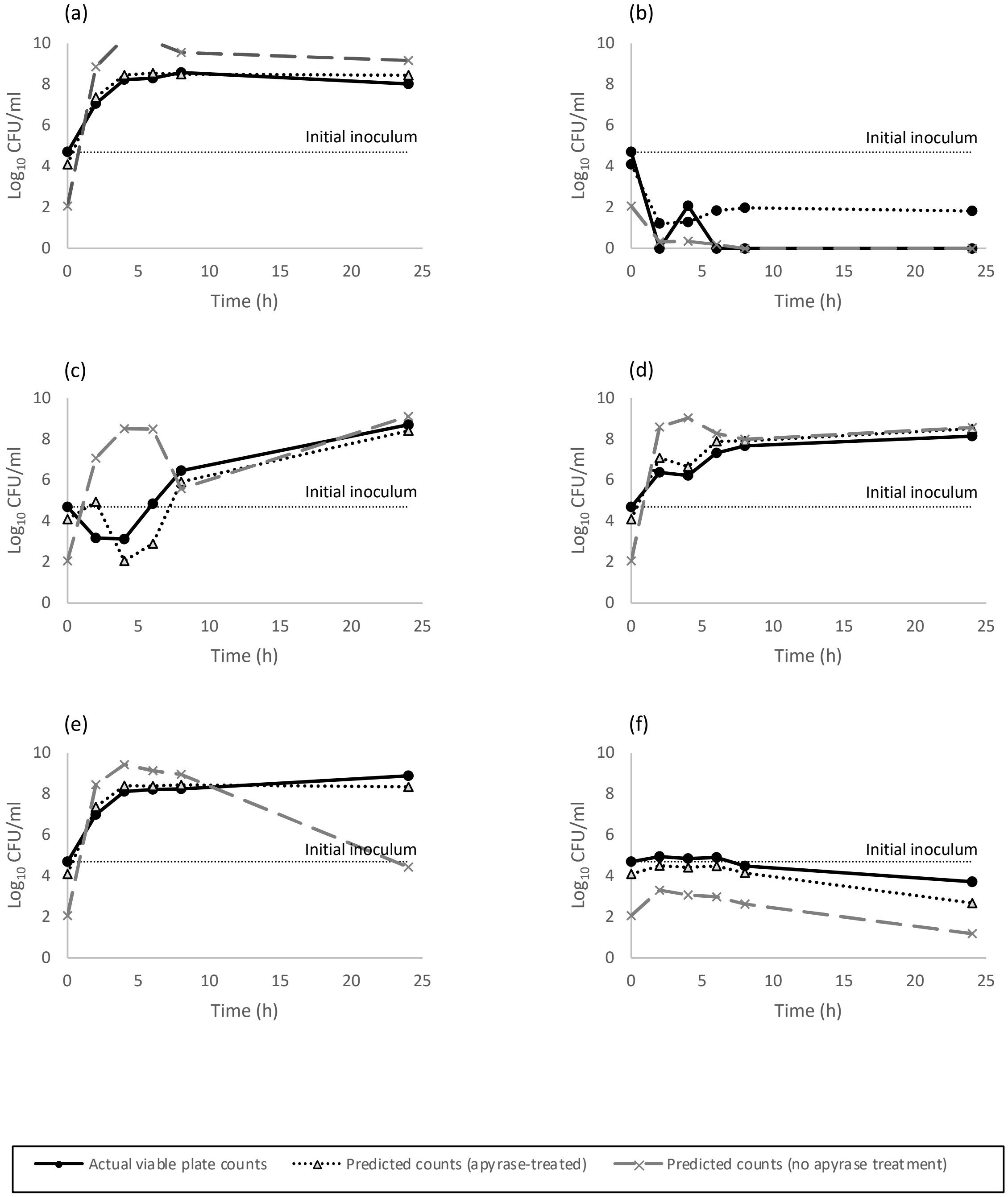

Supplement: Supplementary file 1 [file microorganisms-08-01489-s001.pdf]
